# Editorial: Ophthalmic disease and quality of life

**DOI:** 10.3389/fmed.2023.1178442

**Published:** 2023-04-04

**Authors:** Ioanna Mylona, Mario Damiano Toro, Asimina Mataftsi

**Affiliations:** ^1^Department of Ophthalmology, General Hospital of Serres, Serres, Greece; ^2^Department of Ophthalmology, Federico II University Hospital, Naples, Italy; ^3^Department of Ophthalmology, School of Medicine, Faculty of Health Sciences, Aristotle University of Thessaloniki, Thessaloniki, Greece

**Keywords:** ophthalmic disease, quality of life, health outcomes, PROM (patient reported outcome measures), vision related quality life

Ophthalmic disease affects a large percentage of the population worldwide (1), especially since a number of conditions are very frequent with the advent of time as part of normal aging. Vision loss in ophthalmic disease is associated with increased risk of social isolation, falls and injuries, depression, and other psychological problems. It can amplify the adverse effects of other chronic illnesses, increasing the risk of all-cause and injury-related mortality (2). Moreover, the economic and social costs of vision impairment and eye disease to patients, the healthcare system, and society are considerable and often neglected.

Vision-related quality of life is defined by the World Health Organization as a “complex trait that encompasses vision functioning, symptoms, emotional wellbeing, social relationships, concerns, and convenience as they are affected by vision” (3). While a large number of studies have attempted to present the benefits of providing adequate treatment for an underlying disease for patients' vision-related quality of life, the notion that vision is only a small component of general health and quality of life (4) is only recently being reassessed (5).

This special issue addresses the impact of ophthalmic disease on patients' quality of life and improvements on this metric following treatment, presenting approaches including but not limited to the presentation of clinical data, examinations of the effectiveness and validation of patient-reported outcomes measures (PROMs), and investigation of the general positive impact on health.

In a large epidemiological study, Ye et al. estimate the prevalence of visual impairment in China at 6.22%, determine the geographical variation in prevalence, and identify alcohol intake, hypertension, diabetes, lung diseases, liver diseases, stroke, and heart disease as factors associated with it. This study shows the complex interlinkage between ophthalmic disease and systemic disease that often remains undervalued by clinicians.

In a case–control observational study, Zhmud et al. assess the severity of dry eye disease (DED) and its impact on quality of life in patients with type 2 diabetes mellitus. They find that DED adversely impacts patients' quality of life regardless of the association of the disease with keratopathy. This study shows the additional burden that ophthalmic disease can place on a patient with chronic somatic disease.

Three original studies focus on providing the appropriate standard of care in order to ensure the best future outcomes for patients. Chen et al. retrospectively review 371 cases of unilateral anisometropic amblyopia in which the patients had been treated with patching therapy and followed up for at least 2 years; they detail the course of changes in the refractive error of the amblyopic eye compared to the fellow eye. Wan et al. retrospectively study the course of 116 myopic patients who underwent posterior chamber phakic intraocular implantable collamer lens (ICL) implantation to assess whether superior clear corneal incision (CCI) differs from temporal CCI in terms of avoiding ocular surface inflow into the anterior chamber. They conclude that temporal CCI is preferable to superior CCI in this respect, implying a lower risk of infection and inflammation. Inhalational methanol poisoning can cause severe damage to visual function, and the Shen et al. study of 14 such patients follows up their course. The authors conclude that the risk factors of duration of toxic exposure, delayed admission, and degree of acidosis are strongly associated with the prognosis in terms of visual acuity.

The special issue also includes two case reports: Huang et al. present an 8-year-old child with Weill–Marchesani syndrome and abnormal lens thickening, and Cai et al. present a 44-year-old woman with an intraretinal microcyst with crystalline content and retinal detachment.

We hope that the articles in this special issue will help to draw more attention to the importance of treating ophthalmic disease, not only to the extent that such treatment produces improvement in vision functioning, but also in terms of the general benefits to patients' lives.

## Author contributions

All authors listed have made a substantial, direct, and intellectual contribution to the work and approved it for publication.
